# Involving Family Caregivers in Developing an Intervention for Assessing Risk of Dental Pain in Individuals Living with Dementia

**DOI:** 10.3390/geriatrics10020035

**Published:** 2025-03-05

**Authors:** Carrie Stewart, Nicole Thomas, Robert Witton, Ewen McColl, Patricia Schofield

**Affiliations:** 1 Ageing Clinical and Experimental Research Team, Institute of Applied Health Sciences, University of Aberdeen, Aberdeen AB25 2ZD, UK; 2 School of Nursing and Midwifery, Faculty of Health, University of Plymouth, Plymouth PL4 8AA, UKpatricia.schofield@plymouth.ac.uk (P.S.); 3 Peninsula Dental School, University of Plymouth, Plymouth PL4 8AA, UK; robert.witton@plymouth.ac.uk (R.W.); ewen.mccoll@plymouth.ac.uk (E.M.)

**Keywords:** dementia, dental health services, oral health, caregivers, co-development, public and patient involvement

## Abstract

**Background**: Experiences of dental pain among older people living with dementia, particularly those residing in care homes, have been identified as an under-researched area. There is an urgent need for initiatives, co-developed with experts according to their experience, to address key challenges for oral health improvement among older people living with dementia. This paper reports the findings from a participatory activity which informed the development of an intervention. **Methods**: Informed by discussions with a prominent PPI representative in the field of caring for persons with dementia and a prior multi-disciplinary stakeholder event, a discussion involving ten caregivers of individuals with dementia was conducted. Caregivers were invited from different regions of the UK, with seven joining an online group discussion and three engaging in one-on-one conversations. Transcripts of the conversations based on three topics of discussion (dental experiences, dental challenges and thoughts on a dental pain risk assessment tool) were analysed using thematic analysis to inform a proposed co-developed model of an intervention which can improve dental care for those living with dementia. **Results**: Areas which informed the next phase of discussions and intervention development were access to dental services, lack of alignment between dental care services and health and social care, and low support for carers in how to carry out mouthcare, specific to the type of dementia lived with. Carers felt that preventing, monitoring and managing dental pain should form part of the care package and that it should not be the responsibility of the carer to conduct a dental pain risk assessment. The key recommendation made by carers was the need for a multi-component intervention. This should be flexible according to individual needs and provide education and support to carers to assist with mouthcare, with responsibility for assessing dental pain risk sitting firmly with a nominated professional. **Conclusions**: Our findings from this discussion group with carers of persons living with dementia identified which areas of mouthcare responsibility should be part of the unpaid caring role and which should form part of a healthcare professional role. This shaped a final stakeholder event and facilitated the development of a grant proposal (which includes one of the caregivers as a co-investigator) to test a co-developed intervention for the prevention of dental pain in persons living with dementia.

## 1. Introduction

Dementia significantly impacts oral health due to a combination of cognitive, motor, and behavioural changes that hinder effective oral hygiene and care, affecting both the individual and those responsible for their care [1,2,3]. It is anticipated that many older people living with dementia experience oral health problems including untreated dental pain due to gum disease, tooth decay, dry mouth and mouth sores, impacting quality of life [4]. However, research into dental pain and the tools which can be used to identify pain is still lacking [5]. In 2012, the British Dental Association emphasised the importance of dental care services for residents in care homes, in particular those providing dementia care [6]. A recent report published in March 2023 [7] showed that there has been an improvement in oral health knowledge in care homes, with a 61% to 91% increase in awareness of the NICE oral health guidance (NG48). However, the report highlights the need for more work to ensure all care plans cover oral health, as 40% of staff still had not received oral health training [7].

There is an urgent need for solutions and innovations which can address key challenges in improving the oral health of older people living with dementia. Utilising an iterative process that involves a diverse range of stakeholders is of paramount importance in steering decision-making and fostering innovation [8], even within the realm of oral health. This approach acknowledges the complexity of healthcare systems and the multifaceted nature of health-related challenges, such as those associated with dementia. By engaging multiple stakeholders, especially service users, a comprehensive perspective is achieved, ensuring that a wider spectrum of expertise, experiences, and viewpoints are considered [9]. An iterative approach characterised by continuous feedback loops and incremental improvements facilitates the identification and rectification of shortcomings early in the dementia innovation development cycle [10]. This not only accelerates innovation by refining concepts and strategies but also significantly reduces the risks and costs associated with researching poorly conceived ideas.

**Figure 1:**
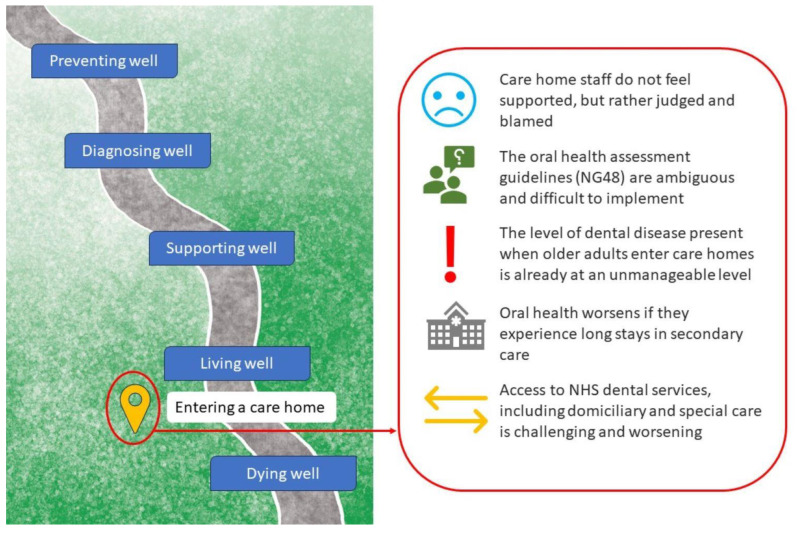
Identified challenges from a preliminary stakeholder event focusing on care home admission in the dementia care pathway which informed the discussion group described in this paper.

In this collaborative stakeholder project, we carried out a series of stakeholder events using a Delphi approach, funded by The Pearn Charitable Trust, to explore potential innovative solutions for the identification of dental pain in those living with dementia [11]. The preliminary event highlighted the need to start the innovation journey earlier in the dementia pathway, which would necessitate including those living with dementia or caring for a person with dementia into the discussions. This was due to a consensus that the level of dental disease and pain already being experienced in the care home negated the need for a risk/prevention tool (see Figure 1). Therefore, the next phase of consultation was to identify difficulties with oral health and mouthcare and to explore how potential solutions might meet the needs of those living with dementia, as well as their role in dental pain risk assessment and prevention. The purpose of this was to inform the next phase of stakeholder discussions and shape a logic model for the development of an intervention intended for an NIHR grant proposal.

## 2. Materials and Methods

### 2.1. Invitation

We distributed an invitation via several routes to promote awareness of the group discussion, including online (https://www.dementiayealm.org/, accessed 4 March 2025). We utilised known professional and Patient and Public Involvement and Engagement (PPIE) networks in the fields of dementia, dentistry and ageing health across the UK. This included advertising on the GUIDE Oral Health platform (https://guideoralhealth.crowdicity.com, accessed 4 March 2025) and with known PPIE groups (e.g., the Oral and Dental Research Patient and Public Involvement (PPIE) group, run by the University of Dundee, https://www.nhsresearchscotland.org.uk/public/help-shape-research/patient-and-public-involvement-groups, accessed 4 March 2025; the Ageing Clinical and Experimental Research PPIE group, https://www.abdn.ac.uk/iahs/research/acer/acer-patient-and-public-involvement-group-1971.php, accessed 4 March 2025). Interested persons were asked to register their intent to join with the research team and were sent an outline version of the discussion topics and a joining link for the meeting. Those intending to attend were informed that discussions would be recorded in order to download a transcript to aid the writing-up process.

### 2.2. Design

One group discussion was held online on the 23rd of November 2022. Consent to record was obtained again. We introduced the topic and gave an overview of the preliminary stakeholder event. On-screen prompts for questions were included, which were the same as those sent in the invitation. Following the overview, attendees were asked to feedback their thoughts on the rationale for talking with them and to discuss a further three topics:

- Dental experiences;
- Issues, challenges and mouthcare concerns;
- Thoughts and opinions about a proposed dental pain risk assessment tool.

The group discussion lasted 90 min. For those unable to make the scheduled group session time or who were unfamiliar or uncomfortable with an online group discussion, an alternative online 1-2-1 session was offered at a time convenient to them throughout November 2022. These 1-2-1 discussions lasted about 60 min and followed the same format as the group session. All those who gave feedback either in the group or 1-2-1 were given a GBP 30 shopping voucher for their time and contributions, including reviewing our summary report.

### 2.3. Analysis

The discussion was recorded and transcribed, with any identifying information removed from the transcript. Recordings were immediately deleted once the transcript had been checked for inaccuracies by NT. Two members of the project team (CS and NT) summarised information under three headings—Challenges, Experiences and Solutions—to reflect the three key topics discussed. Although robust research methods are not necessarily required for co-creative participatory activities, a thematic analysis approach was applied according to Clarke & Braun (2006) [12] which involved both inductive and deductive approaches to theme development. This enabled identification of key themes that would influence the discussion topics for the penultimate stakeholder event and inform the logic model. We did not collect demographic information. A report with our summaries was sent to all attendees for sense checking and final comments to ensure accurate representation of the discussions which would be used to guide the next phase of stakeholder discussions.

## 3. Results

Attendees were made up of paid (*n* = 1) and unpaid carers (*n* = 9). From this point forwards, attendees will be referred to carers.

A total of ten dementia carers attended, with seven taking part in the online group discussion and three carers having 1-2-1 discussions separate from the group discussion. This was due to being unavailable at the time of the group discussion but still being keen to contribute. The carers were either currently caring for a family member with dementia (*n* = 5), had historically cared for a family member with dementia (*n* = 2) or worked professionally in the care industry (*n* = 3). We had representation from across the UK (England Southwest: *n* = 5; England Southeast: *n* = 1; England Northeast: *n* = 1; Scotland: *n* = 3).

There was agreement about involving carers in developing an intervention for preventive measures early in the dementia pathway, including the development of a dental pain risk assessment tool. However, the carers felt that this tool was not intended for unpaid or informal carers to use directly. Carers believed that identifying and monitoring the risk of dental issues and future pain should be the responsibility of healthcare teams, not unpaid carers. They mentioned that their day-to-day challenges left them with little capacity to think about future problems.

To demonstrate how these discussions have therefore influenced our intervention development, we will present summaries under two headings: “Challenges of Mouthcare” and “Exemplars of Mouthcare”. This is followed by a section on how the discussions informed our stakeholder event under “Informing the Logic Model”.

A list of all key themes found is presented in Table 1.

### 3.1. Challenges of Mouthcare

#### 3.1.1. Mouthcare Practices

The multifaceted challenges described by the carers underscored the importance of a holistic, patient-centric intervention. Adaptable strategies involving education, collaboration among caregivers and healthcare teams, and addressing motivational aspects appear crucial for effective prevention of dental pain in individuals with dementia.

Caregivers expressed frustration over the lack of information and support provided, particularly concerning oral health and mouthcare. The intervention should therefore prioritise comprehensive training and education for caregivers, addressing the gap in knowledge about caring for individuals with dementia, with a specific focus on oral health.

Addressing unfamiliarity with dental tools like electric toothbrushes requires incorporating traditional tools and visual aids to minimise confusion. Simplified instructions and hands-on guidance for brushing techniques were described as essential for individuals with difficulties in motor patterns.

For many carers, responding to the willingness and receptiveness of carrying out mouthcare meant that brushing was often a weekly occurrence and not daily. Flexible dental hygiene routines that consider cognitive fluctuations and receptiveness are therefore crucial. Tailoring recommendations and support based on dementia type and the real-time challenges carers are facing was seen as a way to enhance intervention effectiveness by individualising the intervention.

At-home care provision challenges included the fact that limited time allocations, like 15 min blocks, cause distress for those living with dementia who perceived the agency staff as strangers in their home. Concerns were raised about agency staffs’ mouthcare quality, especially when location impacted care time due to uncompensated travel or when specialised strategies were needed. An example was given where a unique physical issue caused limited mouth opening, resulting in deteriorating mouthcare, and led to reduced wellbeing, increased frailty and increased hospitalisations due to falls.

Additionally, information sharing gaps regarding oral health and mouthcare between healthcare providers, care homes and families disrupted coordinated care, leading to incomplete care packages and care instability. Carers highlighted the importance of enhancing communication between dental and healthcare professionals to prevent the need for repetitive information from caregivers. They expressed concerns about their own ability to effectively share crucial information between the two professional services. Questions were therefore raised about the current lack of integration between dental records and general health records, emphasising the need for more cohesive information sharing.

#### 3.1.2. Recognising/Communicating Pain

Discussions identified how the development of an effective intervention necessitates an approach that encompasses communication training, streamlined access to dental services and specialised pain management strategies that acknowledge the intricate interactions between dementia-related challenges and dental discomfort.

Being able to distinguish between behaviours arising from dental discomfort and those stemming from the progression of dementia is paramount. Carers felt that the connection between dementia medications and dry mouth led to potential misinterpretation of related behaviours. Therefore, the intervention should provide education for caregivers and healthcare professionals about medication side effects. Carers who had received information on the potential impact of certain medications found it helpful in understanding unusual behaviours and took steps to mitigate them.

Two further examples of difficulty in communication of pain were given: one of a person expressing the desire to visit a dentist without being able to explain the reason and a second example being a person repeatedly requesting to see the dentist post-treatment because they were aware of pain in their mouth but were unable to recollect the appointment. This emphasised the importance of facilitating non-verbal communication methods that can help individuals convey their pain or discomfort.

Incorporating this into the intervention’s design involves fostering a comprehensive understanding of the unique communication needs of individuals with dementia. Providing caregivers and healthcare professionals with tools to interpret non-verbal cues and address pain-related expressions can contribute to more accurate pain assessment. Simplifying appointment procedures and communication pathways can enhance timely access to dental care. Moreover, designing an intervention that accounts for the complex relationship between memory and pain may improve post-treatment support and overall pain management for individuals with dementia.

#### 3.1.3. Access to Dental Services

Dental examinations provided relief and reassurance for carers and families, with the surveillance of dental issues and management of potential pain by professionals being a comfort to those caring for the person with dementia. The identified barriers discussed by the carers related to physical access and negative childhood experiences impacting their emotional response to dental visits. Additional practical barriers, exemplified by one carer’s efforts to obtain dentures, highlighted the need for the intervention to offer guidance and resources to overcome such challenges.

An intervention should therefore focus on improving access. It should encompass creating dementia-friendly dental environments, providing flexible service delivery and addressing affordability and availability of services. It should include comprehensive training for dental professionals in dementia care and healthcare professionals in mouthcare, with the integration of dental health into overall care plans. A case of a caregiver facing criticism despite their dedicated efforts underscored the importance of empathy and understanding within the healthcare community of the challenges carers face with mouthcare.

The challenges faced by caregivers and care homes providing dementia care in arranging dental services for home visits emphasised the need for flexible service delivery models. Developing intervention strategies that enable dental care to be provided within care home settings or through mobile dental units could enhance access for individuals with mobility issues or those residing in care homes. Collaborations between dental professionals and care home staff should be promoted to ensure the integration of dental health into overall care plans. This was proposed as Oral Health Champions within care homes supporting those living with dementia.

The financial aspect and the variability of available dental services call for an intervention that addresses affordability and service consistency within the confines of the NHS dental contract. Offering a range of services, including hygienist care, could cater to diverse needs. However, lessons from a reported traumatic experience further demonstrate the importance of training dental professionals in providing care that is sensitive to the unique challenges faced by individuals with dementia.

### 3.2. Exemplars of Mouthcare

#### 3.2.1. The Dental Environment

Carers based in Scotland had examples of good practice which addressed some of the challenges highlighted within the discussion. This was thought to be because of the differences in the dental NHS contract in Scotland compared to England: over 60s receive free dental examinations in Scotland, reducing the financial barriers to accessing dental services.

Several carers relayed positive experiences relating to the dental professionals’ knowledge and skills and their understanding of dementia, making reasonable adjustments during assessments. Good support from dental staff, including dentists and hygienists, helped resolve tensions between individuals with dementia and their caregivers regarding dental health behaviours, as those living with dementia were more receptive to the professionals’ advice compared to their loved ones who were perceived as nagging. One participant shared that accessing hygienist services was often easier than accessing dentists, and these services felt more appropriate for prevention, providing reassurance. However, it was acknowledged that not everyone could afford to subsidise hygienist services.

One good practice example involved a clinic offering open sessions for persons with dementia to familiarise themselves with the clinic environment without undergoing examination or treatment. This approach helped address common difficulties attending dental surgeries, such as noise, smells and unfamiliar equipment, which are similar to those experienced by individuals with autism and sensory processing conditions. Another example of good practice was dental professionals performing dental assessments in the care home, which is more commonplace in Scotland. Being in a familiar environment helped those with dementia who had physical impairments making it difficult to leave the care home.

#### 3.2.2. Care Provision

Familiarity with the same service providers was helpful for carers and persons with dementia, as it helped build trust and provided reassurance that needs were being heard and understood. This was particularly important for mouthcare if it was part of the needs assessment. Having at-home care that was for a longer period of time, such as an hour in the morning, helped carers feel at ease that all healthcare and self-care needs had been adequately taken care of so they could get on with the rest of their day. Having less responsibility for these components of care meant that carers could concentrate on wellbeing and enrichment activities.

Care homes for those living with dementia that included mouthcare in their needs assessments provided reassurance to carers that the oral health of their loved ones would be taken care of. Care homes who advertised oral health priorities were more attractive to those who had the choice in choosing a care home. Care homes with Oral Health Champions were seen as best practice.

### 3.3. Developing the Logic Model

**Figure 2:**
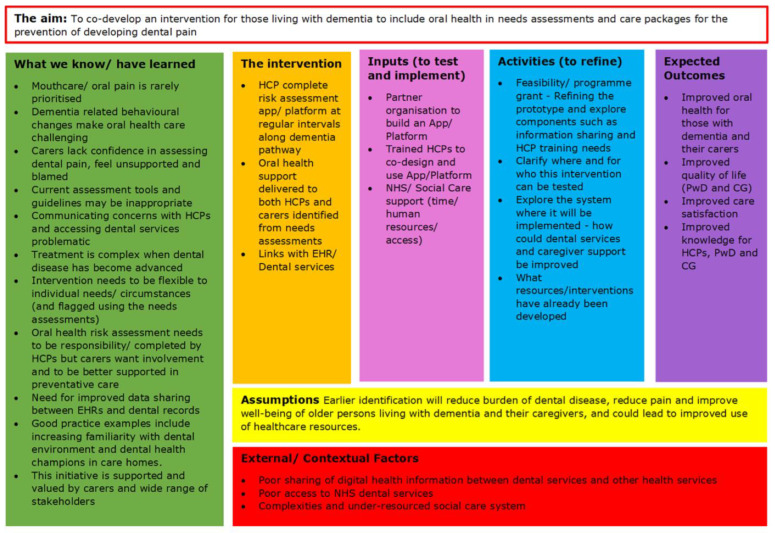
The logic model illustrating considerations for a co-developed oral health intervention for people living with dementia.

The findings from our carer discussions, combined with our work with the preliminary stakeholder event [11], were added to a logic model (see Figure 2), illustrating what we know about the problem, what we still do not know and what action is needed. After agreement with those who participated, this model was taken to our penultimate stakeholder event to seek further consensus on the following:

- **Improving communication pathways:** What are the complexities involved in the integration of patient records with dental records, how interoperable are current electronic record systems used by multiple agencies and what are the feasibility issues relating to a central hub for information dissemination?
- **Upskilling dental and healthcare professionals:** What CPD and training is currently available to professionals coming into contact with those living with dementia, what are the complexities to integrated working and what are the feasibility issues related to upskilling professionals?
- **Responsibility:** Who can take responsibility for identifying dental disease risk and how is it escalated, who can take responsibility for ensuring the upskilling of staff and who can take overall responsibility in overseeing this?

Family caregivers were also asked who they thought should be invited to the next stakeholder meeting to review the logic model. They suggested that more representation from GPs and social workers was needed due to their perceived decrease in mouthcare priorities between primary care and secondary care. The project team acted upon this and invited these professional to the final stakeholder event.

## 4. Discussion

Being informed by the initial outcomes of the preliminary stakeholder event, discussions with carers have guided our intervention development and provided us with invaluable insights, shaping our logic model. We have learned that several good practices are in place, mainly in Scotland. Building on these good examples rather than continuing to identify challenges has helped us avoid ‘reinventing the wheel’ or duplications of research activities.

The key insight is the unanimous agreement that the responsibility for oral health practices, monitoring and management should sit within the paid caring role and be part of personalised care packages. This is not with a view to offload all mouthcare responsibilities to healthcare professionals but to instead focus on family caregiver knowledge and an understanding of how to prevent future dental problems in the presence of other more variable dementia-related behaviours like night snacking or polypharmacy. This has changed the initial direction of this intervention, which was initially focused on a pain assessment tool to be administered by family caregivers.

Additionally, carers informed us that the treatment and support of those living with dementia requires multiple agencies, with the GP at the centre of coordination. They stressed how challenging and complex this was. Recognising and communicating concerns about pain with multiple agencies can be problematic, especially when unpaid carers may not be included in decision-making conversations [13]. Exploring the pathways to better communication between the GP and the multiple agencies involved, including dental services, will be crucial to ensuring the quality of care delivered and received by the person living with dementia. Understanding this and building solutions for these into the proposed intervention allows us to create a more integrated and collaborative oral health system which can meet the needs of all stakeholders.

The representation of carers from across the regions of the UK offered valuable insights into effective care integration. In addition to the good practices already mentioned, it helped us to highlight regional differences that might impede the translation of an intervention across regions. These insights have been integrated into the proposed intervention to ensure that the intervention can be deliverable across regions.

It is noteworthy that, despite our efforts, we did not manage to involve individuals living with dementia in the discussions. It may be that our attempt to hear from individuals across the UK, necessitating remote data collection, may have been a barrier to participation for those with dementia. Remote participation may have been more daunting for those living with dementia. There is also the issue of the topic itself; our carers reported many negative experiences with dental care systems, some of which were reported as being traumatic for the person living with dementia. It may be that discussing dental experiences could be considered a potentially sensitive and distressing topic for those living with dementia. This concern should be explored further so that future efforts in this area can design participatory activities that reflect this. Nevertheless, the stakeholder conversations exhibited a high level of quality, as attested by the richness of information found in the summaries and further illustrated in Table 1.

The feedback from the carers was that the project was important and that they were keen to remain involved as the project developed. The input from caregivers, alongside the other stakeholder events [11], has allowed for a co-developed intervention to be proposed and for further funding to be applied for. It has helped us ensure that our proposed intervention can be meaningful and impactful to those it is designed to support. It has also given voice to the plight of unpaid carers, exhausted by their care duties, which impacts their own oral health and mouthcare practices. The impact upon caregivers themselves was an important addition to the proposed intervention, and as such, quality of life and carer wellbeing as measures of intervention effectiveness have been included.

## 5. Conclusions

We know that pain is “An unpleasant sensory and emotional experience associated with, or resembling that associated with, actual or potential tissue damage” [14], and we are aware that 40% of older adults in the community have poorly controlled chronic pain; this increases to 80% in the care home population and is largely attributed to communication barriers and the high incidence of dementia amongst this group [5]. Sadly, therefore, our most vulnerable in society are most likely to have poorly controlled pain. However, something as simple as effective mouthcare could reduce the amount of pain and suffering of many residents and adults living with dementia.

Our findings from this stakeholder group with carers of persons living with dementia have allowed us to identify critical issues to be considered in the design of a future intervention. These are issues driven from stakeholder events and carers of those living with dementia. The developed logic model can be used to steer the development of future oral health interventions for persons living with dementia.

Many of the challenges highlighted are not new to us and suggest that despite these challenges being documented within the literature nearly twenty years ago [15], very little appears to have been done to translate this into quality improvement and practice. This evidences the need and urgency for innovation in this area and for the involvement of key stakeholders in policy decision-making to identify points of failure in future intervention implementation.

## Figures and Tables

**Table 1:** Themes related to challenges, experiences and solutions around mouthcare for those living with dementia.

Challenges of Present Dental Care	
**Ability to provide mouth care**	Specific challenges for mouthcare were dependant on the individual’s temperament and type of dementia they were living with
	The ability and beliefs of the carer impacted whether or not mouthcare was being attempted
	The affordability of dental care sundries required to meet the mouthcare needs of the individual impacted mouthcare behaviours
**Recognising & communicating pain**	Communicating mouthcare needs were challenging for people with dementia
	Recognising pain in those living with dementia was more challenging
	Effectively communicating needs to emergency dental services may be impacted by the lack of available information from the above two points
**Access to dental services**	Access was impacted by the physical ability of individuals
	Dental services which were appropriate and able to deal with the individual needs were not always located near the individual
	Childhood fears of dentists were triggered by smells causing avoidance
	Arranging for at home visits were an additional burden for carers
	Those accessing private dental services (including hygienists) felt this should be an available resource for all
**Time provided by external support**	Carers felt the amount of time care agency staff were allocated impacted whether mouthcare was being effectively carried out
**Access to helpful educational material and training**	Carers felt they would benefit from training in how to care for their loved ones with dementia, which should include mouthcare
	Educational material delivered compassionately could support them with prioritisation of behaviours, advocacy and decision-making
	Without adequate education, unnecessary treatments or medications might be sought by carers
**Carer burden impacting on carers own dental health**	Persistent advocacy between multiple agencies impacted the mental health and wellbeing of carers
	Poor mental health impacted the carer’s ability to look after their own dental health
	Lack of co-ordination and information sharing between care provisions perpetuating the challenges facing carer advocacy
**Positive experiences and exemplars**	
**The Dental Environment**	Having familiarity with a welcoming dental environment was helpful to accessibility
	The knowledge of dental professionals was useful in order for reasonable adjustments to be made
	Being able to access hygienist services (availability and affordability) was seen as important for preventative mouthcare
**Care Provision**	Mouthcare being part of needs assessments was reassuring to carers
	Receiving knowledge on factors which could impact mouthcare was valued
	Familiarity of care providers helped build trust
**Carers advocacy skills**	Advocacy is a big part of caring duties which was seen as a particular skillset needed in order to navigate the health and social care system
**Good Practices**	Dental practices providing dementia-friendly sessions to help build familiarity was seen as good practice
	Having domiciliary dental care within care homes helped inform decisions about choosing a care provision
	Having longer a singular block of care provision may be of more benefit that smaller blocks of care throughout the day
	Oral health champions within care homes provided reassurance about the mouthcare priorities of the care home
**Attributes of potential solutions**	
**Flexible to reflect the complexities**	Interventions need to carefully consider both the complexities of the different types of dementia as well as the complexities of the health and social care system
**Increase carer knowledge and skills**	The intervention for carers should be focused on supporting carers in improving knowledge and skills by up-skilling the health and social care system in mouthcare
**Responsibility for risk assessments**	Dental risk assessments should be the responsibility of the health and social care system so carers can concentrate on the day-to-day challenges
**Information sharing**	Linking up EHRs with dental records to facilitate better coordination of care

## Data Availability

The datasets presented in this article are not readily available because the data was collected as part of Public and Patient Involvement and Engagement activities. Requests to access the datasets should be directed to carrie.stewart@abdn.ac.uk.
